# Molecular Rearrangement in Aromatic Amino Acids and Proteins After Reaction with Hydroxyl and Hydroperoxyl Radicals and UV-C Radiation

**DOI:** 10.3390/molecules30204046

**Published:** 2025-10-10

**Authors:** Irina Ivanova, Igor Piskarev

**Affiliations:** 1 Department of Molecular Biology and Immunology, Lobachevsky Nizhny Novgorod State University, 603950 Nizhny Novgorod, Russia; ivanova.ip@mail.ru; 2 Skobeltsyn Institute of Nuclear Physics, 119234 Moscow, Russia

**Keywords:** aromatic amino acids, free radicals, proteins, quenching and igniting fluorescence, Stern–Volmer coefficient

## Abstract

The fluorescence of aqueous solutions of the aromatic amino acids tryptophan, tyrosine, and phenylalanine, an albumin solution, and a mixture of water-soluble animal and plant proteins is investigated after treatment with hydroxyl and hydroperoxyl radicals and continuous UV-C radiation at λ = 253.7 nm. The use of independent sources of active species allows for the study of activation and the development of free radical processes in model objects. The analysis is based on Stern–Volmer coefficients for the quenching of the fluorescence of the initial substrates and the ignition of the fluorescence of newly formed products. In the reaction with hydroxyl radicals, the hydrogen atom could be abstracted from any position in the target molecule. Under continuous UV-C radiation, the protein molecule as a whole was excited.

## 1. Introduction

Modification of proteins by radicals affects their structure and functions. The influence of radicals on various metabolic substrates and biologically important molecules determines the direction of cell metabolism of products (apoptosis, phagocytosis, etc.). Protein modification is assessed by the fluorescence of aromatic amino acids. During the process of metabolism, active forms of oxygen are produced in the body, among which hydroxyl radicals play a major role. Free radicals are primarily generated during oxidative stress. Therefore, the channels of interaction with free radicals are considered additionally. After interacting with hydroxyl radicals, protein radicals are formed, which mainly convert into peroxyl radicals and hydroperoxides. Hydroperoxides of proteins are formed in cells and tissues during oxidative stress [1]. Reactions with radicals have a significant impact on the development of various pathological processes and require additional efforts for antioxidant protection [2].

Detailed studies on the effects of UV radiation on proteins have been conducted for a long time [3,4,5]. However, the mechanisms of photoaging are still being researched, and the effects of photo-modification on proteins and other molecules, cells, and organisms are relevant to this day. Reactions under the influence of UV radiation proceed through type I and II mechanisms, forming secondary active species that interact locally with large organic molecules or through a mechanism in which a molecule absorbs a photon as a whole. Comparing these mechanisms is an important task.

In study [6], the ability of pterin to induce structural and chemical changes in HSA under UV-A irradiation was investigated. Results showed that pterin is able to photoinduce oxidation of the protein in at least two amino acid residues: tryptophan and tyrosine. This is a specific reaction pathway; UV-A radiation has a very low probability of generating free radicals. The products formed during the degradation of amino acids and proteins involving pterin are fundamentally different from the products formed during interactions with free radicals, as these are different reaction pathways.

The kinetics of oxidation of amino acids, a dipeptide, and BSA by two persistent water-soluble free radicals of the hydrazyl type have been studied [7]. The oxidation rates of the studied products were compared, explaining the role of reactive intermediates in biological systems as well as in work dedicated to environmental remediation [8].

The importance of protein oxidation under the influence of active external environmental factors, including RONS and UV radiation, in the aging processes of organisms is considered in reference [9].

The authors of [10] summarize the results of studies designed to elucidate basic mechanisms of reactive oxygen-mediated oxidation of proteins and free amino acids. Study [11] studied the formation of fragments that occur during the oxidation of proteins and dipeptides under the action of hydroxyl radicals.

In the practice of biochemical research, methods play an important role where selective influence on specific points of metabolism in biological objects is possible. One area of research is the interaction of free radicals with biological samples. Free radicals are generated in the body during the process of metabolism. The hydroxyl radical can form all types of reactive oxygen species during subsequent chemical transformations [12]. The study of radical processes in the body and the metabolism of individual radicals remains a complex task. Currently, it is possible to generate various active forms of oxygen using specially designed devices. Devices that create free radicals include various types of plasma generators [13]. The composition of free radicals has a wide range and depends on the composition of the working gas. Such devices do not possess selectivity.

A generator of ozone-hydroxyl mixture which allows for the treatment of samples with hydroxyl radicals has been developed [12]; a generator of pulsed hot plasma radiation, which creates hydroperoxyl radicals in the liquid samples, has also also developed [14]. The use of these devices allows for the investigation of reactions with a specific radical.

The aim of this study is to study and analyze the mechanisms of fluorescence of aromatic amino acids and proteins of animal and plant origin in reactions with hydroxyl and hydroperoxyl radicals and under the influence of continuous UV-C radiation using the Stern–Volmer coefficient.

## 2. Experimental Results

### 2.1. Fluorescence of the Initial Products

Characteristics of active species sources used in this work, see in Table 1.

The values of excitation and registration wavelengths, obtained experimentally in this work for all initial products, as well as for substances that may have formed during destruction under the influence of active species, are presented in Table 2.

Detailed data is in the Materials and Methods Section.

For the amino acids tyrosine, tryptophan, and phenylalanine, the fluorescence yield reaches a maximum at specific values of excitation and registration wavelengths, as shown in Table 2 (Section 4). The characteristics of the fluorophores were measured directly in this work.

**Figure 1:**
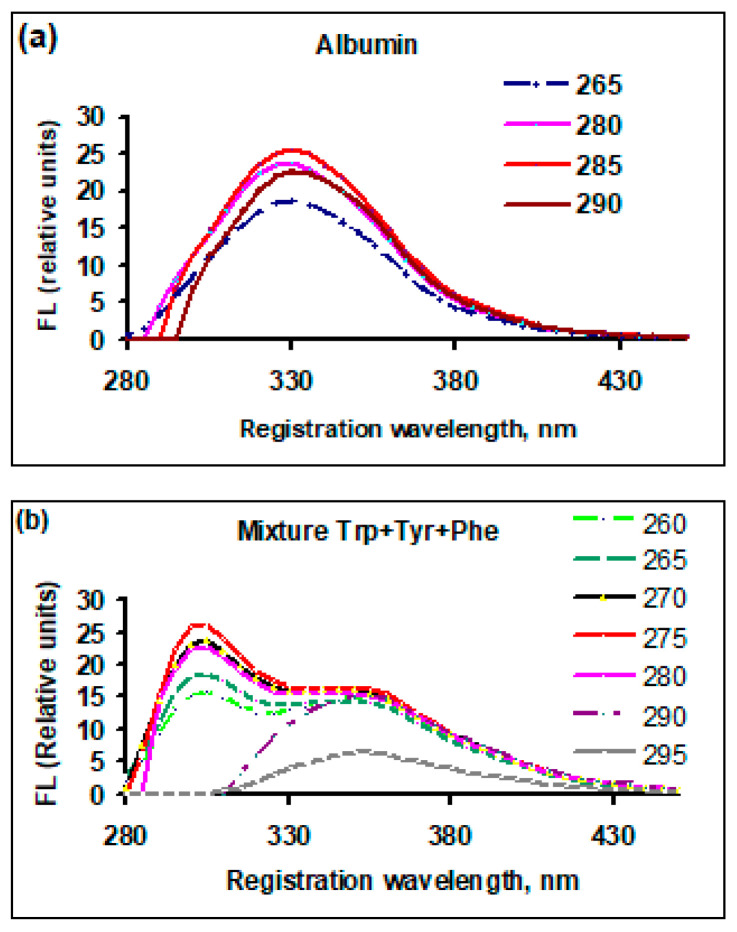
The dependence of the fluorescence yield of the initial (untreated) solutions at different excitation wavelengths, FL (relative units), on the registration (emission) wavelength, nm: (**a**) bovine serum albumin; (**b**) a mixture of amino acids contained in bovine serum albumin, with the same concentration ratio as in the native albumin molecule. The numbers on the right side of each graph are excitation wavelengths, nm.

The fluorescence of amino acids in bound and free states within the protein albumin and in a mixture of amino acids in a solution with an amino acid, in which the ratio of its concentration was similar to that in albumin, was studied at different excitation wavelengths. The results are presented in Figure 1.

For albumin (Figure 1a), when changing the excitation wavelength, the fluorescence yield changes, but the shape of the peak and the position of the maximum registration wavelength remain unchanged. In a mixture of free amino acids (Figure 1b), the fluorescence yield has two peaks, which is characteristic of tyrosine and tryptophan (registration wavelengths of 305 and 350 nm). With increasing excitation wavelength, when tyrosine levels cannot be excited (290 and 295 nm), only the fluorescence peak of tryptophan (~350 nm) remains. The observed features can be explained by the difference in the fluorescence mechanisms of amino acids in their free state and as part of a protein.

**Figure 2:**
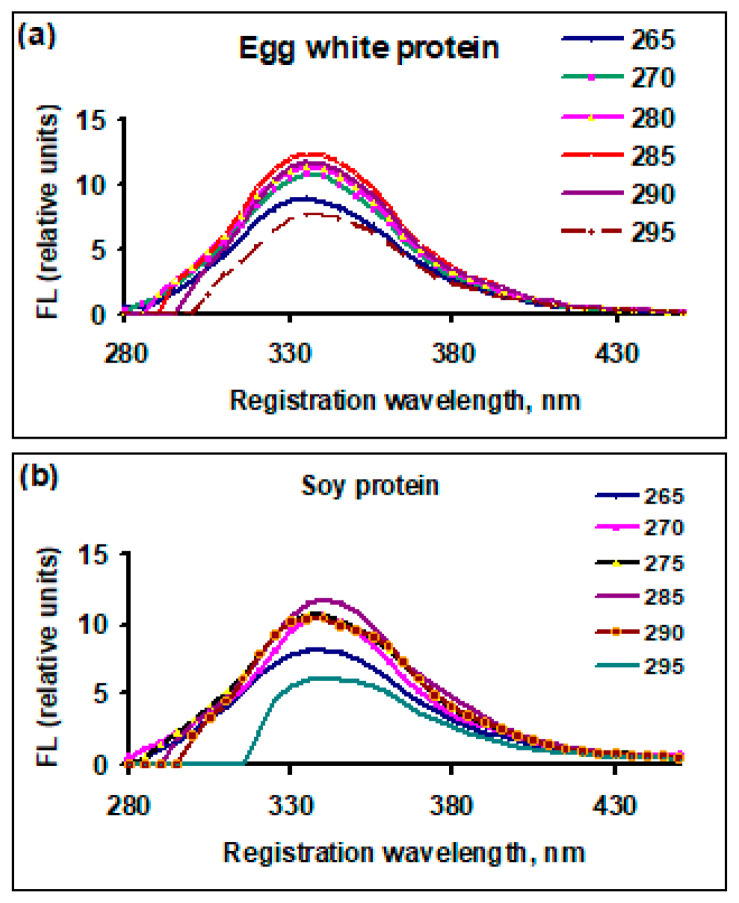
Dependence of the fluorescence yield of the initial (untreated) protein solutions at different excitation wavelengths, FL (relative units), on the registration (emission) wavelength, nm: (**a**) egg white protein; (**b**) soy bean protein. The numbers to the right of each graph: excitation wavelengths, nm.

The fluorescence of solutions of egg and soy bean protein at different excitation wavelengths is shown in Figure 2.

For both types of protein, the maximum emission (yield) of fluorescence was achieved at the same excitation wavelength (285 nm). The position of the fluorescence emission maximum did not depend on the excitation wavelength and was 335 nm for egg white protein and 340 nm for soy bean protein.

### 2.2. Fluorescence of Treated Solutions

**Figure 3:**
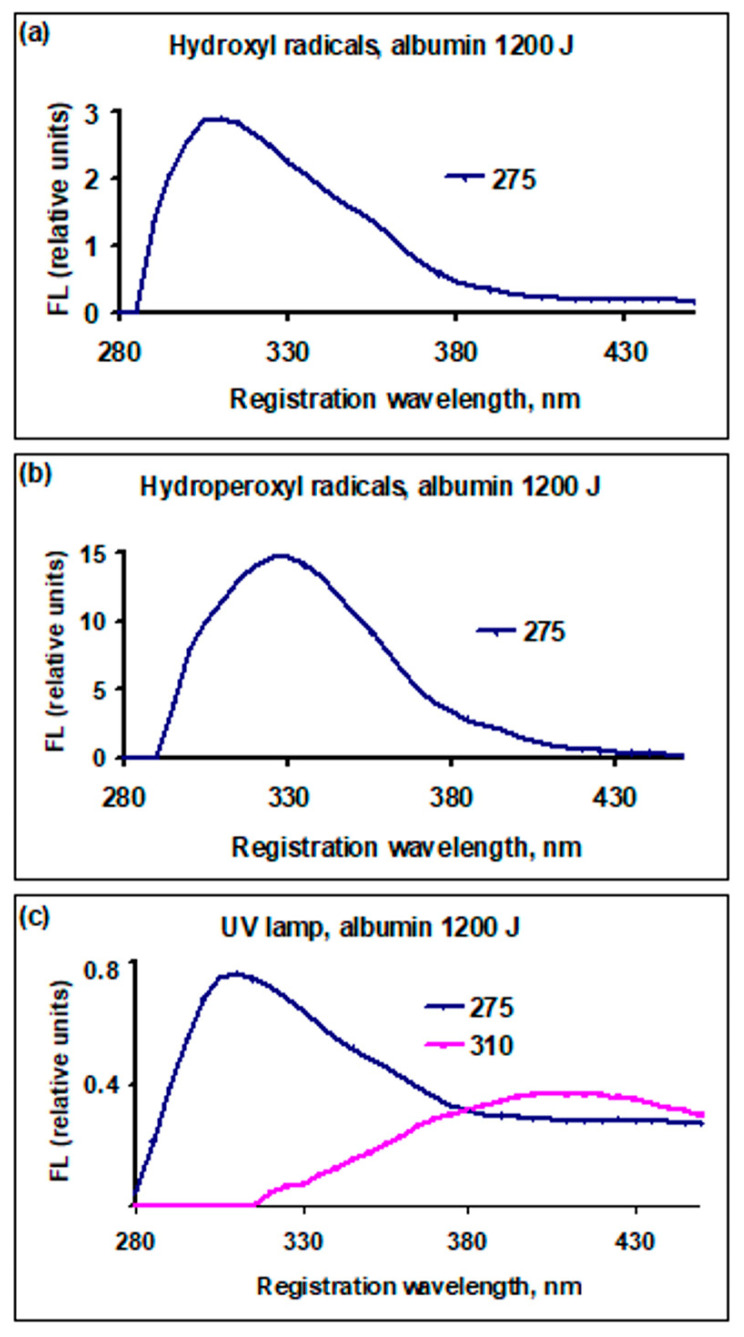
Dependence of the fluorescence yield of bovine serum albumin solutions, FL (relative units), on the registration (emission) wavelength, nm, treated with active species, dose 1200 J (10 mL)^−1^: (**a**) cold plasma generator, ^•^OH radicals; (**b**) hot plasma generator, radicals $HO_{2}^{•}$ ; (**c**) UV lamp radiation λ = 253.7 nm. The numbers on the right side of each graph: excitation wavelengths, nm. The red line—the formed dityrosine.

**Figure 4:**
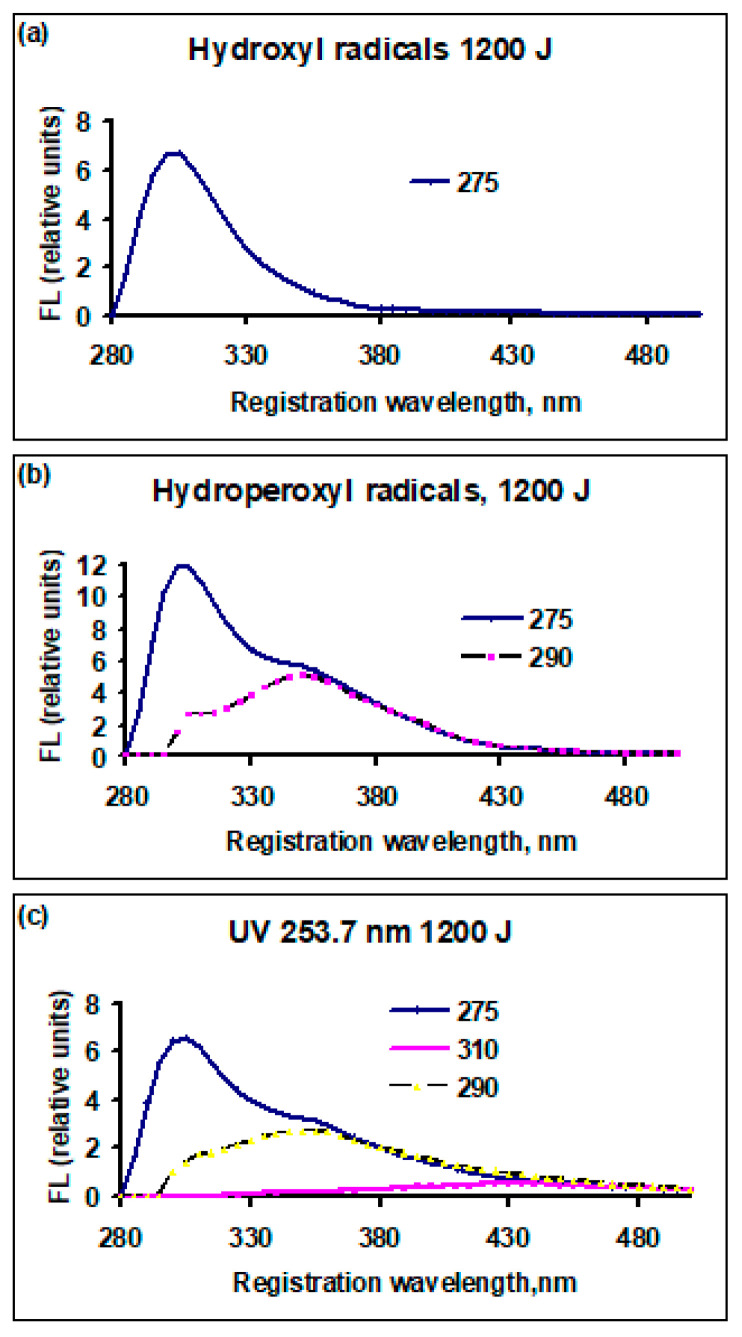
The dependence of the fluorescence yield of amino acid solutions, similar in composition to bovine serum albumin, FL (relative units), on the registration (emission) wavelength, nm, treated by active species, dose 1200 J (10 mL)^−1^: (**a**) cold plasma, radicals ^•^OH; (**b**) pulse plasma radiation, radicals $HO_{2}^{•}$ ; (**c**) UV radiation λ = 253.7 nm. The numbers on the right of each graph: excitation wavelengths, nm.

The dependence of fluorescence yield for bovine serum albumin and an albumin-like amino acid mixture, after exposure to active species with a dose of 1200 J (10 mL)^−1^, is presented in Figure 3 and Figure 4. In all cases, the fluorescence yield decreased compared to the initial value.

In the albumin solution after treatment with hydroxyl radicals using a plasma generator (Figure 3a), the maximum wavelength emission position shifted from 330 to 310 nm. After treatment with hydroperoxyl radicals, the maximum position did not change. Under the influence of UV lamp radiation, the maximum shifted to 310 nm and a line appeared at the excitation wavelength of 310 nm with a maximum at the registration wavelength of 410 nm. The shift in the maximum was due to the rapid consumption of tryptophan compared to tyrosine and the decrease in the contribution of tryptophan to the total yield.

In Figure 4, the excitation wavelength of 275 nm corresponds to the emission maximum. With the increase in the excitation wavelength, part of the fluorescence channels is closed. At λ_ex_ = 290 nm, it is impossible to excite tyrosine; at λ_ex_ = 310 nm, it is impossible to excite tyrosine and tryptophan. This is related to changes in the fluorescence spectrum. In Figure 3, λ_ex_ = 275 nm is taken to be the same value as in Figure 4 to exclude any possible dependence of the emission maximum position on λ_ex_. Although the experiment showed that, when λ_ex_ corresponded to the maximum emission, wavelength position λ_reg_ does not change, the amount of emission was no more than 10% at λ_ex_ = 275 nm less than the maximum possible at the optimal value of λ_ex_.

In the albumin-like mixture after treatment with ^•^OH radicals (Figure 4a), a peak remained at an excitation wavelength of 275 nm, as was the case for the non-treated solution, and a registration wavelength of 305 nm. After treatment with $HO_{2}^{•}$ radicals (Figure 4b), in addition to the peak at 305 nm, a peak was observed at an excitation wavelength of 290 nm and a registration wavelength of 340 nm. Under the influence of UV radiation (Figure 4c), the situation was similar to that in Figure 4b, but a new broad peak appeared at an excitation wavelength of 310 nm and a registration wavelength of 410–450 nm, which can be attributed to the isomer of dityrosine. In the region, there was an excitation wavelength of 325 nm and a registration wavelength of 434 nm which was attributed to NFK (N-formylkynurenine) [6]; no appreciable fluorescence yield was observed.

The characteristics of dityrosine were studied in study [15]. Dityrosine was detected by means of measuring the absorption spectra, and there was a peak at 280 nm. The excitation wavelength of dityrosine fluorescence ranged from 285 to 320 nm, depending on the pH of the solution. Fluorescence excitation spectra of dityrosine were monitored at wavelengths of λ_reg_ = 380–450 nm [15]. The dependence of λ_ex_ on the pH value in an alkaline medium was established [15], but in the pH range from 2 to 7.5, (in this study, pH = 3) the value of λ_ex_ corresponding to the fluorescence maximum did not change.

**Figure 5:**
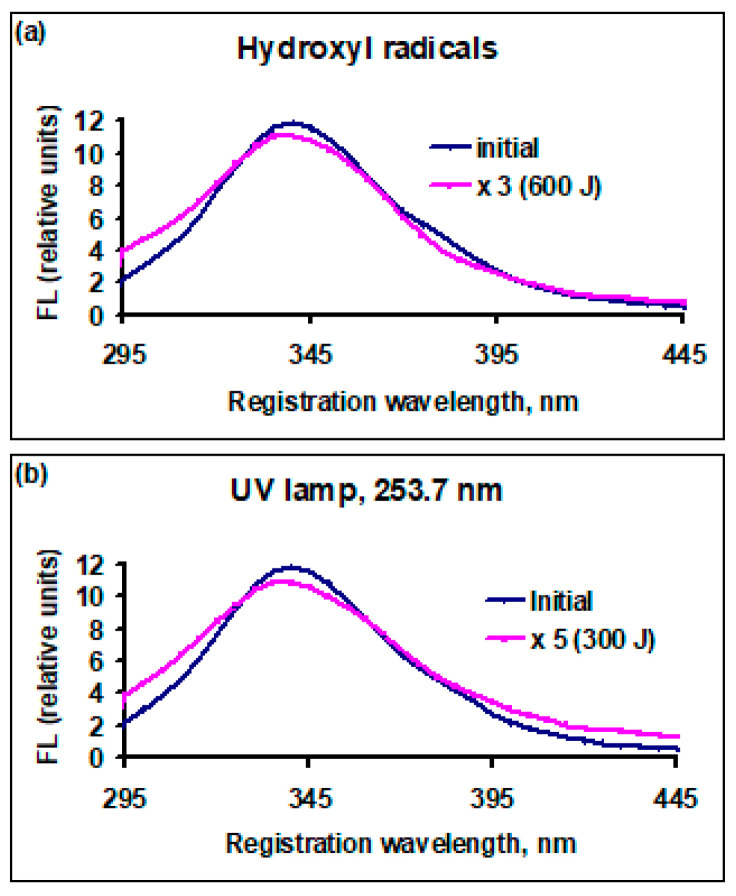
Dependency of fluorescence yield, FL (relative units), for soy bean protein solutions on registration (emission) wavelength before and after treatment with active species: (**a**) cold plasma, ^•^OH radicals, initial solution before exposure (black line) and after exposure to radicals (red line, FL × 3), dose 600 J (10 mL)^−1^; (**b**) UV radiation 253.7 nm, initial solution before exposure (black line) and after exposure to radiation (red line, FL × 5), dose 300 J (10 mL)^−1^.

In soy bean and egg white proteins, the position of the maximum wavelength of registration does not change, the fluorescence yield decreases with increasing dose, and the appearance of new peaks was not observed. Figure 5 shows the dependence of the fluorescence yield on the wavelength of registration for a solution of soy bean protein treated with plasma (hydroxyl radicals) and UV lamp radiation as an example. With further increases in the dose, the position of the fluorescence maximum did not change and the emission decreased.

Thus, it has been established that, under the action of active species, the fluorescence of amino acids in their free state and those that are part of proteins is quenched. During the breakdown of amino acids and proteins, new fluorescent products are formed. In their free state, amino acids fluoresce independently; by selecting the excitation wavelength, the contribution of a specific amino acid can be selected.

When amino acids are part of a protein, the protein molecule fluoresces as a single entity, making it impossible to select the contribution of individual amino acids. The change in the pH of the solution within the range of 2.5 to 7.5 leads to a change in the intensity of fluorescence, but the position of the fluorescence maximum does not change and the separation of the contributions of individual amino acids is not observed.

### 2.3. Stern–Volmer Coefficients

The Stern–Volmer coefficients for all investigated observed products of the reactions are presented separately in Table 3 for fluorescent amino acids in Table 4 for proteins.

## 3. Discussion

### 3.1. Initial State of Proteins

The fluorescent products that are part of proteins are tryptophan, tyrosine, and phenylalanine. The fluorescence yield of phenylalanine is low [15], so the fluorescent agents in the protein albumin are tryptophan and tyrosine. In a mixture of proteins of animal and plant origin, in addition to tryptophan and tyrosine, there may be degradation products of amino acids that possess fluorescent properties. The excitation and registration wavelengths at which the fluorescence yield is at its maximum differ significantly for tryptophan and tyrosine, see Table 2. Therefore, in the mixture of amino acids at different excitation wavelengths, one can observe registration maxima corresponding to tryptophan and tyrosine (Figure 1b). From this, it can be concluded that, in the mixture of amino acids, each amino acid is excited and fluoresces independently. In solutions of proteins of the same composition (Figure 1a) or a mixture of animal and plant proteins (Figure 2a,b), the maximum fluorescence yield was observed at practically the same registration wavelengths (330–335 nm) and does not depend on the excitation wavelength, provided that the energy of the excitation photon is greater than the energy of the fluorescent level. If the amino acids are within the same protein, then due to intramolecular energy transfer [16], the emission bands overlap, and one line is emitted. Depending on the ratio of concentrations of tryptophan and tyrosine in a single protein, the position of the emission line may change, but in a mixture of a large number of different proteins, the emission line averages out (Figure 2).

### 3.2. Characteristics of Active Species and Their Ability to Interact with Amino Acids and Proteins

The experiment involves three types of active particles: ^•^OH, $HO_{2}^{•}$ radicals, and continuous UV radiation. Let us consider each of these factors separately.

#### 3.2.1. ^•^OH and $HO_{2}^{•}$ Radicals

In the reaction of organic compounds with radicals, the first stage involves the abstraction of a hydrogen atom from the target molecule [17].

R-H + ^•^OH → R^•^ + H_2_O (1)

(2) $$\mathrm{R-H}+HO_{2}^{•}\to R^{•}+H_{2}O_{2}$$

Here R^•^ is any organic radical that includes fragments of amino acids or proteins. In these reactions, energy is released, which can be used to detach a hydrogen atom from the target molecule. In Reaction (1), about 480 kJ mol^−1^ of energy is released, while in Reaction (2) it is about 368 kJ mol^−1^. The bond energy of the hydrogen atom connected to the α-carbon of the aromatic amino acid is 360–380 kJ mol^−1^. For the other carbon atoms, the C-H bond energy is 430–450 kJ mol^−1^ [18]. Thus, the hydroxyl radical can abstract a hydrogen atom from almost any position in the target molecule, while the hydroperoxyl radical can predominantly detach a hydrogen atom from the α-carbon atom. In Reactions (1) and (2), the radical R^•^ is formed, further transformations of which determine the composition of the products initiated by Reactions (1) and (2). Thus, radicals locally interact with certain sites of amino acids that are part of proteins, such as C-H and C-C.

#### 3.2.2. UV Photons of Continuous Radiation 253.7 Nm

The mechanisms of interaction with matter of continuous UV-C range radiation passing through air (200–280 nm) have been thoroughly analyzed over a long period of time [14]. There are monographs dedicated to these studies [19,20]. One of these mechanisms is the direct absorption of photons by chromophores that have energy levels in the UV-C range. A chromophore that absorbs a photon can transit to an excited state or transfer the excitation to the entire molecule, after which a disruption of the molecule’s structure may occur. Another mechanism involves type I and type II reactions with initiators (photosensitizers) in the presence of oxygen dissolved in water. A review of photodestruction processes under the influence of UV radiation has been published [21,22,23]. In type I reactions, a water-soluble photosensitizer ^1^S in the singlet ground state under UV-C radiation generates the singlet excited state ^1^S*, which undergoes intersystem crossing (ISC) to form the triplet excited state ^3^S*. Then ^3^S* interacts with oxygen to form an ion–radical, $O_{2}^{•-}$ (Reactions (3) and (4)).

The triplet state ^3^S interacts with substrate S and with a molecule of oxygen, which is also in the ground triplet state, under the action of a photon. As a result, an ion–radical, $O_{2}^{•-}$, is formed.

(3) S1→hνS*1→ISCS*3

(4) S*3+O23→O2•−3

In a type II reaction, a photosensitizer in a triplet or singlet state reacts with a molecule of oxygen, which is in the ground triplet state, under the influence of a photon. As a result, singlet oxygen, ^1^O_2_, is formed [24].

(5) S3+O23→hνS1+O21

(6) S1+O23→hνS3+O21

Photodestruction via type I and II reactions has been experimentally observed in a large number of studies. However, for the mechanism of photodestruction through chromophores to be noticeable, the light intensity must be high, the power of the lamp must be around 300 watts, and the processing time should be around 10 to 20 h [25].

Single amino acids can be photosensitizers. Nitrites can serve as photosensitizers, as the N-O bond of nitrite ions dissolved in water can be broken under the action of UV-C photons, but the yield of such a process is very small [21,25].

(7) $$NO_{2}^{-}+h\nu \to NO^{•}+O^{•-}$$

Under the influence of UV-C radiation, hydroxyl radicals ^•^OH can be formed in water. The mechanism for the formation of hydroxyl radicals in water can be the following process:

(8) H2O+hν→O•H+H•

The dissociation energy of the H-OH bond in a water molecule is 485 kJ mol^−1^ [18]. This corresponds to the energy of a photon of 5.03 eV or a wavelength of λ = 246 nm. However, the probability of such a process is low [26]. There is a significant probability that this process will occur under radiation with a wavelength of less than 190 nm [27], but radiation of such wavelength does not pass through air. For this process to occur, the quartz mercury lamp must be fully submerged in water.

Study [28] shows that, under the influence of light radiation, hydrogen peroxide can be formed in water. At the first stage, an excited water molecule is formed.

**Scheme 1:**
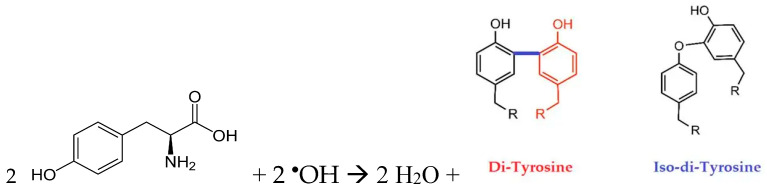
Structure of the forming dityro-sine isomers.

In water saturated with dissolved oxygen, Scheme 1 is possible:

(9) $$H_{2}O^{•}+O_{2}\to H_{2}O_{2}+1/2O_{2}$$

The range of wavelengths at which Reaction (9) is possible is from 200 to 1220 nm [28]. Thus, the formation of hydrogen peroxide in water is possible not only under the influence of UV radiation but also across the entire light spectrum and partially in the IR range. The formation of hydrogen peroxide under the influence of daylight and red light radiation was experimentally observed in study [29].

The impact of UV radiation on aqueous solutions of amino acids and proteins can occur through local interaction of intermediate particles with the structural components of the protein, i.e., with amino acids (type I or II reactions), or due to the direct absorption of photons by the entire protein molecule. In the absorption spectrum of any water-soluble protein, there is a line with a maximum at 260–280 nm, so direct photon absorption in this range is possible. The energy of a UV-C photon with a wavelength of 253.7 nm (low-pressure mercury lamp) is 470 kJ mol^−1^. Absorbing such energy will lead to the excitation of the entire protein molecule. Due to intramolecular redistribution, energy can be transferred, including to aromatic amino acids. Tyrosine, tryptophan, and phenylalanine are capable of fluorescence. The intensity of fluorescence of aromatic amino acids depends on their concentration, and if the amino acids are degraded, the yield of fluorescence decreases. When the protein in the cell is destroyed, new products may appear, such as Schiff’s bases—azomethines, whose structural formula is R^1^R^2^C = NR^3^, which will fluoresce in their excitation and registration wavelength range.

The fluorescent characteristics of proteins are determined by the contribution of tryptophan and tyrosine. Non-aromatic amino acids that are part of proteins, such as histidine, methionine, cysteine, and cystine (disulfide bonds), can also absorb energy from radiation in the UV-C range. By redistributing through intramolecular transfer, energy can be concentrated at the level of fluorescent molecules. Amino acids that are part of a single protein molecule can be considered as coupled oscillators emitting energy at a single wavelength. This wavelength will depend on the contribution of each oscillator. When the contributions of individual oscillators change, the emitted wavelength will also change. That is, in the composition of the protein, tyrosine and tryptophan will emit one line, the wavelength of which will depend on the relative concentrations of tyrosine and tryptophan. The contribution of phenylalanine will be small due to the low yield of fluorescence. The bond energies of C-H and C-C in the aromatic ring are between 350 and 450 kJ mol^−1^ [18]. Thus, under all the influences applied in this study, the aromatic rings of amino acids can be destroyed. This may result in the destruction of the entire protein molecule, leading to the formation of both fluorescent and non-fluorescent residues.

### 3.3. Destruction of Aromatic Amino Acids

To analyze the mechanism of destruction of aromatic amino acids and to determine the contribution of various reaction pathways, we use the Stern–Volmer coefficient $K_{sv}^{0}$. The coefficient $K_{sv}^{0}$ characterizes the degree of influence of a specific physical factor on the fluorescence of the substance under study, which creates a dose, D, in the solution. The probability of interaction between the target substance and the active species is proportional to the magnitude of the Stern–Volmer coefficient, provided that each active species creates the same dose. This allows for comparison of different factors. For the reactions of aromatic amino acids with radicals and UV radiation, the coefficients $K_{sv}^{0}$ are presented in Table 3.

#### 3.3.1. Tryptophan

The highest value of the Stern–Volmer coefficient was achieved during the treatment of the sample with hydroxyl radicals, $K_{sv}^{0}$ = 24 × 10^−3^ (10 mL) J^−1^. Under the influence of UV radiation, the value was approximately 5 times lower, and the smallest value, 0.7 × 10^−3^ (10 mL) J^−1^, of the coefficient was observed during the treatment of tryptophan under the influence of $HO_{2}^{•}$ radicals. It was previously assumed [18] that the main cause of the degradation of tryptophan is the removal of a hydrogen atom from the α-carbon atom, which may lead to the formation of fluorescent residues. For hydroperoxyl radicals, the removal of hydrogen from the α-carbon atom, based on the energy bond estimates mentioned above, should be the main reaction pathway. However, the value of $K_{sv}^{0}$ in the reaction with hydroperoxyl radicals was 34 times lower than that for hydroxyl radicals and 6 times lower than for UV radiation. In addition, no fluorescent reaction residues were observed. This means that reactions with hydroperoxyl radicals play a minor role, and the products of this reaction were not observed. Under the influence of hydroxyl radicals, hydrogen atoms were removed from practically any position in the tryptophan molecule. At the same time, fragments of the molecule were formed that did not contain an aromatic or indole ring. The formation of fluorescent residues was not observed even under UV radiation. Therefore, the destruction pathway involving the removal of a hydrogen atom from the α-carbon atom in tryptophan did not play a significant role. There is data supporting this conclusion [6].

#### 3.3.2. Tyrosine

The situation here is similar. According to the values of $K_{sv}^{0}$ (Table 3), the detachment of a hydrogen atom from any position in the tyrosine molecule by OH^•^ radicals was 15 times more likely than from the α-carbon atom by the $HO_{2}^{•}$ radicals. The additional product formed during interactions with hydroxyl radicals and under the influence of UV radiation was dityrosine. In both cases, the magnitude of the coefficient $K_{sv}^{0}$ for the fluorescence increased (ignition); for the resulting dityrosine, it was approximately the same. The reaction of tyrosine with hydroxyl radicals (Scheme 1) can lead to the formation of two isomers of dityrosine with excitation wavelengths of 290 and 310 nm at a registration wavelength of 420 nm. In study [14], the formation of dityrosine was attributed to two acid–base forms. However, in this study, the acidity of the solution did not change during the treatment, so the formation of different forms of dityrosine can be ascribed to different reaction mechanisms.

In the reaction with hydroxyl radicals and under the influence of UV radiation during the degradation of tyrosine, an isomer of dityrosine with an excitation wavelength of 290 nm was formed. As can be seen from the structure of the forming dityrosine isomers (Scheme 1), in both cases, there was no detachment of a hydrogen atom from the α-carbon atom. This is consistent with the value of the coefficient $K_{sv}^{0}$ in the reaction with $HO_{2}^{•}$ radicals, which is four orders of magnitude smaller than for the reactions with hydroxyl radicals or UV radiation (Table 3). Tyrosine has an absorption band at 274 nm, close to the emission wavelength of a mercury lamp. Therefore, the excitation mechanism of the tyrosine molecule as a whole via UV radiation absorption is also possible, and according to the values of the coefficient $K_{sv}^{0}$, it has approximately the same probability as the reaction with a hydroxyl radical.

#### 3.3.3. Phenylalanine

The degree of quenching of phenylalanine fluorescence by radicals and UV radiation was approximately the same (Table 3). The secondary fluorescent products were tyrosine and dityrosine. Since the value of $K_{sv}^{0}$ in the formation of tyrosine and dityrosine in the reaction with UV radiation is approximately 10 times greater than in the reactions with radicals, it can be stated that they are predominantly formed upon the absorption of a UV photon by the phenylalanine molecule as a whole.

In the sequential transformations phenylalanine → tyrosine → dityrosine, in which tyrosine is the secondary product, an isomer of dityrosine was formed with an excitation wavelength of 310 nm. In study [25], when phenylalanine was destroyed by sunlight, the formation of 18 products lacking fluorescence properties was observed. This confirms that the destruction reaction of phenylalanine by UV-C radiation is not related to the abstraction of a hydrogen atom from the α-carbon atom but proceeds through a stage of excitation of the entire molecule.

### 3.4. Destruction of Proteins

#### 3.4.1. Albumin

The values of the coefficient $K_{sv}^{0}$ for proteins are presented in Table 4. For the albumin solution, the most effective quenching of fluorescence, indicating the degradation of the original product, occurred under the influence of UV radiation, $K_{sv}^{0}$ = 26 × 10^−3^ (10 mL) J^−1^, while under the action of radicals, the value of $K_{sv}^{0}$ was 20 times lower. Dityrosine was formed only under the influence of UV radiation; the fluorescence ignition coefficient $K_{sv}^{0}$ for dityrosine was equal to 30 × 10^−3^ (10 mL) J^−1^, which is similar in magnitude to the quenching coefficient for the albumin molecule. That is, the greatest changes occurred in the reaction with UV radiation.

Study [30] investigates the formation of -SH groups in albumin under the action of $HO_{2}^{•}$ radicals and UV radiation at 253.7 nm. The yield of –SH groups was found to be five times higher under the influence of UV radiation than in the reaction with radicals. That is, in all studied cases, the process of protein destruction under the influence of UV-C radiation prevailed. The data presented above allows us to say that the main mechanism of interaction of albumin with UV-C radiation is the absorption of a photon and the excitation of the albumin molecule as a whole. The absorbed energy can concentrate on specific fragments of a protein molecule such as tryptophan and tyrosine, or on -SS groups. Unlike UV-C radiation, radicals locally detach hydrogen atoms from a large protein molecule, not only from aromatic but also from other amino acids. There are many more non-fluorescent amino acids in the protein than there are fluorescent ones. Therefore, the main products of albumin degradation are non-fluorescent protein fragments. The energy expended on reactions with any amino acids cannot be transferred to aromatic amino acids. Thus, only a portion of the energy absorbed by the protein from the source of active species in the form of radicals is used to quench the fluorescence of tyrosine and tryptophan. At the same time, UV radiation excites the entire protein molecule, and the excitation is transmitted to individual fragments, among which are tryptophan and tyrosine. The amount of energy absorbed by the whole protein molecule and transferred to aromatic amino acids depends on the properties of the protein.

If fluorescent amino acids are present in the same protein, the emission bands overlap, and a single line is emitted. The destruction rates of amino acids (coefficients $K_{sv}^{0}$) under the influence of different active species differ; some are consumed faster than others. As a result, the position of the maximum of the total peak shifts depending on the type of active species involved.

Such a situation occurred in the case of the destruction of the albumin protein (Figure 4). In the initial albumin solution, the maximum fluorescence occurred at a registration wavelength of 335 nm. After the reaction with active species, the position of the fluorescence maximum shifted towards shorter wavelengths. This is related to the fact that, in tyrosine, the fluorescence maximum is at a shorter registration wavelength than in tryptophan, and the magnitude of the coefficient $K_{sv}^{0}$ for tryptophan is much greater than that for tyrosine. The position of the maximum fluorescence shifted towards shorter wavelengths, as tryptophan was consumed more quickly and its contribution to the overall peak decreased.

#### 3.4.2. Albumin-like Mixture of Amino Acids

In the mixture of amino acids, tryptophan + tyrosine + phenylalanine, where the molar concentration ratios of the amino acids were the same as in the protein albumin, each amino acid fluoresced independently, with two observed emission lines caused by tryptophan and tyrosine (Figure 4). The fluorescence of phenylalanine was low and not noticeable. The values of $K_{sv}^{0}$ for both types of radicals and continuous UV radiation were approximately the same (Table 4). However, the number of tryptophan molecules was much lower than that of tyrosine. Therefore, in the process of destruction of the dissolved mixture, tryptophan was consumed faster than tyrosine; the emission line of tryptophan disappeared, leaving only the emission line of tyrosine.

#### 3.4.3. Protein Blends of Different Compositions

This case was implemented in protein solutions: soy bean protein isolate and egg white protein. As seen in Figure 5, the yield of fluorescence decreased with increasing doses of, or energy carried by, the radicals and UV-C radiation, but the position of the maximum fluorescence registration did not depend on the excitation wavelength in the initial and treated solution and did not change after treatment with active species. This means that the fluorescence of individual proteins is additive—it is integral. The curves describing the dependence of fluorescence intensity on the registration wavelength for different excitation wavelengths were averaged, and one maximum was observed, the position of which did not change.

Thus, it was established that, under the action of active species, the fluorescence of aromatic amino acids is extinguished, both in their free state and as part of proteins. During the breakdown of amino acids and proteins, new fluorescent products may be formed.

In a free state, amino acids fluoresce independently. By selecting an excitation wavelength characteristic of a specific amino acid, it is possible to analyze the contribution of an individual amino acid. If amino acids are part of a protein, the protein molecule fluoresces as a single object, and it is impossible to isolate the contribution of an individual amino acid.

## 4. Material and Methods, Experimental Section

### 4.1. Sources of Active Species

**Figure 6:**
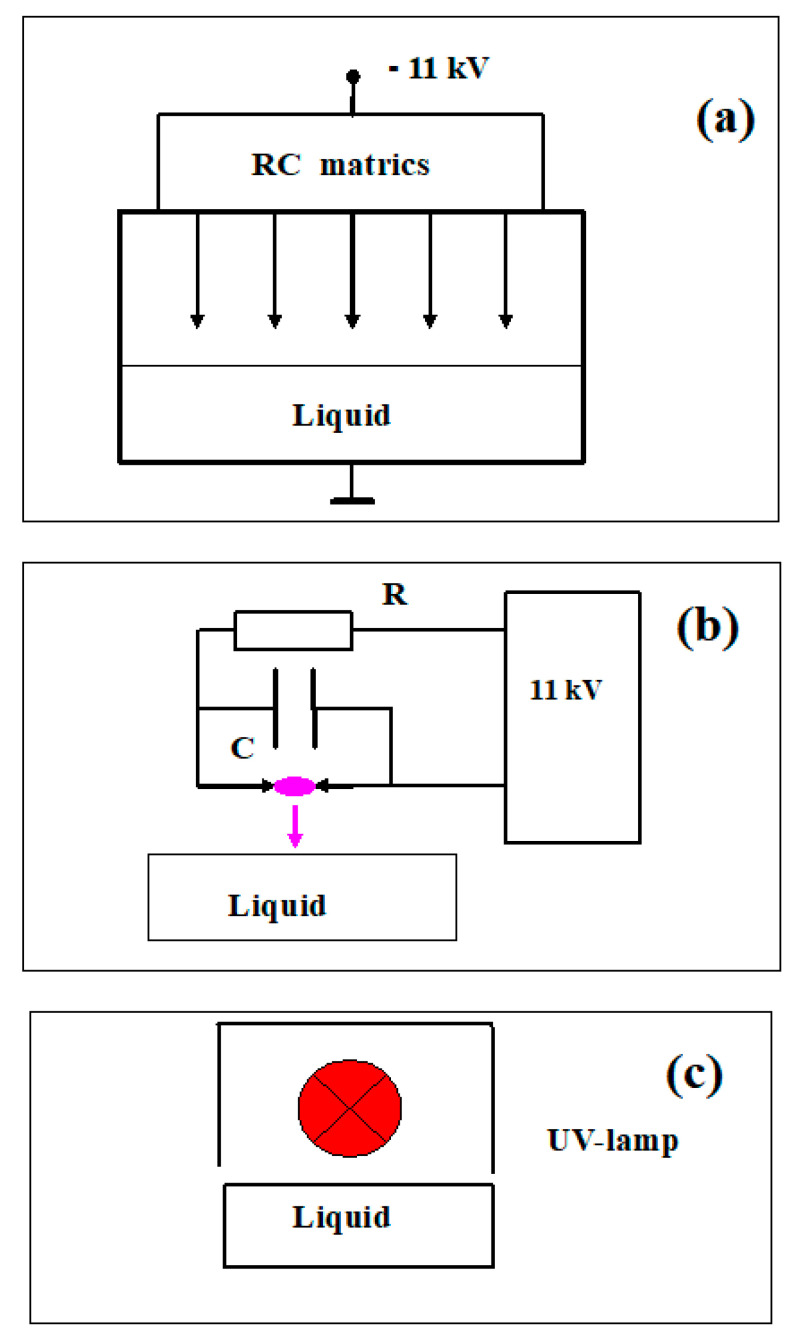
Sketches of experimental setups for processing samples with active species generated by (**a**) cold plasma from corona electric discharge (^•^OH radicals); (**b**) pulsed radiation from hot plasma spark electric discharge ($HO_{2}^{•}$ radicals); (**c**) low-pressure mercury UV lamp, continuous radiation at λ = 253.7 nm.

The sources of active species were a cold plasma generator of corona electric discharge (^•^OH radicals), a pulsed radiation generator of hot plasma from spark electric discharge ($HO_{2}^{•}$ radicals), and continuous radiation from a low-pressure mercury UV lamp (UV-C range photons, λ = 253.7 nm). The experimental setup is shown in Figure 6. A brief description of the devices is provided in study [14]. The characteristics of the sources of active particles are presented in Table 1. The uniqueness of our approach to the investigated problem is that we can separately compare the effect of each factor, ^•^OH and $HO_{2}^{•}$ radicals and UV radiation, in the C-range on the studied substrate.

### 4.2. Object of Research

The research objects were aqueous solutions: the amino acids tryptophan (Trp), tyrosine (Tyr), phenylalanine (Phe), a mixture of the amino acids Trp+Tyr+Phe, bovine serum albumin protein, a complex mixture of animal proteins and chicken egg white proteins (egg white), and a complex mixture of plant proteins and soy bean proteins (soy protein). The fluorescence of the studied substances was measured before and after treatment with active species at a certain concentration depending on time (dose). The fluorescence spectra were recorded using the two-dimensional scanning method. The excitation wavelength range was 260–350 nm, with a step of 5 nm. The range of registration wavelengths was 260–500 nm, with a step of 5 nm. Measurements were carried out using the Fluorat-02 Panorama spectrofluorimeter (Lumex Company, St. Petersburg, Russia). The fluorescent products that were directly part of proteins were tryptophan, tyrosine, and phenylalanine. The quantum yield and extinction coefficient of phenylalanine were low, so phenylalanine was usually not observable [15]. Fluorescent degradation products of amino acids and proteins in the cell included dityrosine, Schiff’s bases, dopamine, and L-DOPA (L-3,4-dihydroxyphenylalanine). The excitation and registration wavelengths of the substances considered are given in Table 2. For dityrosine, the regisration wavelength was 410–420 nm, and in different experiments, maxima were observed at excitation wavelengths of 290 or 310 nm. This case can be regarded as the formation of dityrosine in different isomeric states.

### 4.3. Sample Processing Procedure

Freshly prepared aqueous solutions of amino acids L-tryptophan at a concentration of 20 mg/L (9.8 × 10^−5^ mol/L), L-tyrosine at 20 mg/L (1.1 × 10^−4^ mol/L), and L-phenylalanine at 220 mg/L (1.33 × 10^−3^ mol/L) and bovine serum albumin at a concentration of 700 mg/L (1.07 × 10^−5^ mol/L) were processed. The concentration of the reagents was chosen to be sufficiently low so that the self-absorption of radiation in the sample could be neglected [15]. The criterion was the reduction of fluorescence yield when diluting the sample. In this case, the fluorescence was proportional to the concentration of the substance in the sample. Calibration curves were built that linked the concentration of amino acids and fluorescence yield. A mixture of fluorescent amino acids present in albumin was prepared, in which the ratio of the number of amino acid molecules was proportional to the number of residues of these amino acids in albumin: tyrosine:tryptophan:phenylalanine (20:2:27) [31,32,33]. The concentrations taking into account the molecular weight of each amino acid were tyrosine 20 mg/L, tryptophan 2.25 mg/L, and phenylalanine 24.5 mg/L. During the processing with hydroperoxide radicals, the pH value of the solution decreased, since nitrous acid was formed [12] which led to a decrease in the yield of fluorescence [12,34]. Therefore, sulfuric acid was added to the solutions to stabilize them to a pH level of 3. When the pH value changed, the fluorescence yield changed, but the position of the emission maximum (registration wavelength) did not change. During the processing, the pH of all test solutions did not change. Reagents of analytical grade from the company Laverna (Moscow) and distilled water with a pH of 6.5 were used.

Soy bean protein isolate (from the company "Kompanion City", St. Petersburg, Russia) was dissolved in water. Egg white protein was extracted directly from chicken eggs and prepared in an aqueous solution. The concentration of proteins was set in such a way that, when diluting the solutions, the yield of fluorescence decreased almost linearly with the decrease in concentration. In this case, self-absorption in the sample could be neglected [15,35]. The concentration of the sample for further analysis was not important, as the Stern–Volmer quenching–ignition coefficient was used, which, as will be shown later, does not depend on the concentration of the fluorescent substance. The Stern–Volmer coefficient was used to describe the process of fluorescence quenching after the introduction of substances that suppress fluorescence [36].

### 4.4. Processing of Results

#### 4.4.1. Stern–Volmer Coefficient

To describe the process of static quenching of fluorescence that occurs when substances that suppress fluorescence are introduced into a solution, the Expression (1) was proposed firstly in the work of Stern–Volmer, which was made in 1919. After this coefficient was broadly used [36].

(10) $$\frac{I_{0}}{I_{Q}}=1+K_{sv}[Q]$$

where [Q] is the concentration of the quencher, and I_0_ and I_Q_ are the fluorescence intensities of the solution sample before and after the introduction of the quencher, respectively. The attenuation of fluorescence depends on the concentration of the quencher [Q] and the value of the coefficient, which is commonly referred to as the Stern–Volmer coefficient. It characterizes the degree of impact of the quencher on the sample.

At low concentrations of fluorescent substances, when self-absorption of the emitted radiation in the sample can be neglected, fluorescence is proportional to the concentration of the substance. Under the influence of reactive oxygen species or other factors, the initial substance may be consumed, leading to a decrease in its fluorescence, or a new substance may form, which will increase its fluorescence. Let us consider the possibility of describing the action of active species using an expression similar to (11) with the application of the Stern–Volmer coefficient. Instead of the concentration of the quencher, we take the dose D. We will discuss the relationships for this coefficient in cases where the substance is either consumed or formed in a reaction with active species.

#### 4.4.2. Case: The Substance Is Consumed

In the first stage, when little of the initial material has been consumed, the law of diminishing fluorescence can be expressed as

(11) $$F_{D}=F_{0}e^{-DK_{sv}^{0}}$$

where F_0_ is the fluorescence of the initial sample,

F_D_ is the fluorescence of the same sample after reaction with active species, dose D.

$K_{sv}^{0}$ is the initial value of the Stern–Volmer coefficient at dose D ~0.

Let us consider the following approximate relationship:

(12) $$\frac{F_{0}}{F_{D}}=\frac{F_{0}}{F_{0}\cdot e^{-DK_{sv}^{0}}}=e^{DK_{sv}^{0}}\sim 1+DK_{sv}^{0}$$

Ratio (13) is fulfilled at low doses, $DK_{sv}^{0}<0.1$, and allows us to find the value of $K_{sv}^{0}$.

#### 4.4.3. Case: The Substance Is Formed

Let us consider the case in which the substance is formed and accumulates, and the fluorescence increases. There is a background fluorescence F_0_ in the sample. Then at low values of $DK_{sv}^{0}$,

(13) $$F_{D}=F_{0}e^{DK_{sv}^{0}}$$

(14) $$\frac{F_{D}}{F_{0}}=e^{DK_{sv}^{0}}\sim 1+DK_{sv}^{0}$$

In this case, one can neglect the consumption of the substance being formed under the action of active species, since the concentration of the substance at the initial moment is low. Expressions (13) and (14) are identical; they characterize the dependence of the fluorescence yield of the sample on the properties of the RONS source, which is proportional to the dose (the concentration of radicals and treatment time), both in the case of consumption of the initial substance and the formation of a new substance. The dimensionality of the coefficient $K_{sv}^{0}$ is the relative change in the fluorescence of the product normalized to the dose per unit volume of the sample. In our experiment, the dimensionality used is (10 mL) J^−1^. The coefficient $K_{sv}^{0}$ characterizes the degree of impact of the physical factor on the fluorescence of the investigated substance, creating the dose D in the solution. 

## 5. Conclusions

The channels and the mechanisms of reactions of hydroxyl and hydroperoxyl radicals and continuous UV-C radiation with aromatic amino acids and proteins have been studied. The initial stage of the reaction with hydroxyl radicals involves the abstraction of a hydrogen atom from almost any position in the amino acids and in the protein.

The initial stage of the reaction with hydroperoxyl radicals is the removal of a hydrogen atom from the α-carbon atom. This mechanism plays a minor role in the destruction of aromatic amino acids.

Continuous radiation of the UV-C range is absorbed by a protein as a whole object. Due to intramolecular transfer, excitation energy concentrates on individual channels, leading to the destruction of aromatic amino acids and a decrease in fluorescence yield.

Aromatic amino acids in an unbound, free state fluoresce independently, but if they are part of a protein or proteins, it is impossible to isolate the contribution of an individual amino acid to the overall fluorescence.

The methods of radical generation applied in this study allow for the isolation and investigation of a specific pathway of the reaction initiating the destruction of proteins and amino acids.

## Figures and Tables

**Table 1:** Characteristics of active species sources.

Characteristic	Source of Active Species		
	Corona Electric Discharge	Hot Plasma Radiation	Mercury Lamp
Sample volume, mL	50	10	10
Power of dose, J (10 mL)^−1^ min^−1^	30 ± 3	35 ± 3	20 ± 2
Primary species that carry energy	Electrons E = 10 keV	Photons, pulse radiation λ = 220 nm	Photons, continuous radiation λ = 253.7 nm
Flow of primary species, min^−1^	1.87 × 10^17^	3.9 × 10^19^	2.5 × 10^19^
Type of secondary active species	Radicals ^•^OH	Radicals ${HO}_{2}^{•}$	Photons λ = 253.7 nm
Flow of secondary active species mol (L s)^−1^	(2.1 ± 0.3) × 10^−6^	(1.2 ± 0.3) × 10^−6^	(5.4 ± 0.2) × 10^−8^

**Table 2:** The values of the excitation and registration wavelengths at which the fluorescence of the sample is maximal. Dityrosine, registration wavelength λ_reg_ = 410–420 nm, can exist in two modifications with the following excitation wavelengths: λ_ex_ = 290 nm and λ_ex_ = 310 nm.

Sample	Wavelength, nm	
	Excitation	Registration
Albumin	275	330
Tyrosine	275	303
Tryptophan	285	355
Phenyllanine	260	285
Dityrosine	290, 310	410–420
Dopamine	280	315
L-DOPA	280	320
Schiff’s base	290	455
Egg white protein	285	335
Soy protein	285	340

**Table 3:** Stern–Volmer coefficients, $K_{sv}^{0}$, (10 mL) J^−1^, for fluorescence quenching of the consumed products and ignition of fluorescence of the new formed products for aromatic amino acids as the target. Dityrosine can be formed in two isomeric states at excitation wavelengths of 290 and 310 nm. The error of all data is 10%.

Target	Fluorescent Product	Active Species	Fluorescence $K_{sv}^{0}$ (10 mL) J^−1^	
			Quenching	Ignition
Tryptophan	Tryptophan	^•^OH	24 × 10^−3^	-
	E_ex_ = 285 nm	HO_2_^•^	0.7 × 10^−3^	-
	E_reg_ = 355 nm	UV, 253.7 nm	4.5 × 10^−3^	-
Tyrosine	Tyrosine	^•^OH	4.5 × 10^−3^	-
	E_ex_ = 275 nm	HO_2_^•^	0.3 × 10^−3^	-
	E_reg_ = 303 nm	UV, 253.7 nm	1.4 × 10^−3^	-
	Dityrosine	^•^OH	-	13 × 10^−3^
	E_ex_ = 290 nm	HO_2_^•^	-	<1 × 10^−6^
	E_reg_ = 420 nm	UV, 253.7 nm	-	7.8 × 10^−3^
Phenylalanine	Phenylalanine	^•^OH	1.1 × 10^−3^	-
	E_ex_ = 260 nm	HO_2_^•^	1.4 × 10^−3^	-
	E_reg_ = 285 nm	UV, 253.7 nm	1.3 × 10^−3^	-
	Tyrosine	^•^OH	-	0.7 × 10^−3^
	E_ex_ = 275 nm	HO_2_^•^	-	<1 × 10^−6^
	E_reg_ = 303 nm	UV, 253.7 nm	-	9.5 × 10^−3^
	Dityrosine	^•^OH	-	0.75 × 10^−3^
	E_ex_ = 310 nm	HO_2_^•^	-	<1 × 10^−6^
	E_reg_ = 420 nm	UV, 253.7 nm	-	9.7 × 10^−3^

**Table 4:** Stern–Volmer coefficients, $K_{sv}^{0}$, (10 mL) J^−1^, for the quenching of the fluorescence of consumed products and the ignition of fluorescence of formed new products for proteins as the target. Dityrosine can be formed in two isomeric states at excitation wavelengths of 290 and 310 nm. The error of all data is 10%.

Target	Fluorescent Product	Active Species	Fluorescence, $K_{sv}^{0}$ (10 mL) J^−1^	
			Quenching	Ignition
Bovine serum albumin	Albumin	^•^OH	1.1 × 10^−3^	-
	E_ex_ = 275 nm	HO_2_^•^	1.6 × 10^−3^	-
	E_reg_ = 335 nm	UV, 253.7 nm	26 × 10^−3^	-
	Dityrosine	^•^OH	-	<1 × 10^−6^
	E_ex_ = 310 nm	HO_2_^•^	-	<1 × 10^−6^
	E_reg_ = 420 nm	UV, 253.7 nm	-	30 × 10^−3^
Albumin-like mixture of aromatic amino acids	Tyrosine	^•^OH	1.4 × 10^−3^	-
	E_ex_ = 275 nm	HO_2_^•^	1.2 × 10^−3^	-
	E_reg_ = 305 nm	UV, 253.7 nm	2.2 × 10^−3^	-
	Dityrosine	^•^OH	-	3.1 × 10^−3^
	E_ex_ = 310 nm	HO_2_^•^	-	<1 × 10^−6^
	E_reg_=420 nm	UV, 253.7 nm	-	15 × 10^−3^
Protein blend egg white (animal protein)	Protein E_ex_ = 285 nm E_reg_ = 335 nm	^•^OH	1 × 10^−3^	-
		UV, 253.7 nm	5.1 × 10^−3^	-
	Dityrosine	UV, 253.7 nm	-	3.7 × 10^−3^
	E_ex_ = 310 nm			
	E_reg_ = 420 nm			
	Schiff’s base	UV, 253.7 nm	-	3.7 × 10^−3^
	E_ex_ = 330 nm			
	E_reg_ = 435 nm			
Soy bean isolate protein blend (plant protein)	Protein E_ex_ = 285 nm E_reg_ = 340 nm	^•^OH	3.6 × 10^−3^	-
		UV, 253.7 nm	5.7 × 10^−3^	-
	Schiff’s base	UV, 253.7 nm	-	2.3 × 10^−3^
	E_ex_ = 330 nm			
	E_reg_ = 435 nm			

## Data Availability

Data are contained within the article.

## Abbreviations

RONS	Reactive Oxygen and Nitrogen Species
BSA	Bovine Serum Albumin
HSA	Human Serum Albumin
λ_ex_	Excitation Wavelength, nm
λ_reg_	Emission Wavelength, nm
ISC	Intersystem Crossing
NFK	N-formylkynurenine
